# Predictive model of the risk of formation of mental deadaptation in chemical production workers

**DOI:** 10.1192/j.eurpsy.2022.1767

**Published:** 2022-09-01

**Authors:** S. Kuzmina, R. Garipova, N. Mollaeva

**Affiliations:** 1 Kazan Stae Medical University, Psychiatry, Kazan, Russian Federation; 2 Dagestan Medical State University, Psychiatry, Makhachkala, Russian Federation

**Keywords:** chemical production, mental maladjustment, prenosological diagnostics

## Abstract

**Introduction:**

According to WHO, 30-50% of workers experience psychological stress, which contributes to the development of mental and psychosomatic disorders. It’s important to develop the problem of prenosological diagnosis and determination of criteria for the formation of mental maladaptation as predictors of mental disorders in relation to occupational risk factors. Mental disorders in workers in a dangerous environment are of the nature of prenosological, not clearly delineated by a circle of diagnostic symptoms.

**Objectives:**

1st chemical factor in combination with noise and emotional stress, 2d chemical factor in combination with a threat to life (explosion and fire hazard), 3d working in conditions of noise and emotional stress

**Methods:**

The influence of working environment conditions and non-production factors on the formation

**Results:**

Predicting the development of mental mailadaptive state model was developed

**Conclusions:**

Vegetative disorders are most likely to manifest in group 1, comorbid of which with obsessive-phobic disorders and depressive spectrum disorders. The group 2 is characterized by astheno-vegetative symptoms in correlation with the conversion type of response. In the third professional group, the nature of predictors is characterized by a pronounced isolation of groups of symptoms, the leading of which is the vegetative pattern.Vegetative syndroms are most likely to manifest in group 1, comorbid of which is obsessive-phobic disorders and depressive spectrum disorders. The group 2 is characterized by astheno-vegetative symptoms in correlation with the conversion type of response. In the third professional group, the nature of predictors is characterized by a pronounced isolation of groups of symptoms, the leading of which is the vegetative pattern.

**Disclosure:**

No significant relationships.

